# Whole-Transcriptome Sequencing and Differential Expression Analysis of the Epididymis in Junggar Bactrian Camels Before and After Sexual Maturity

**DOI:** 10.3390/biology14070760

**Published:** 2025-06-25

**Authors:** Jiahao Liu, Xinkui Yao, Jun Meng, Jianwen Wang, Yaqi Zeng, Linling Li, Wanlu Ren

**Affiliations:** 1 College of Animal Science, Xinjiang Agricultural University, Urumqi 830052, China; ljh072412@163.com (J.L.); yxk61@126.com (X.Y.); junm86@sina.com (J.M.); wjw1262022@126.com (J.W.); xjauzengyaqi@163.com (Y.Z.); lilinling@xjau.edu.cn (L.L.); 2 Xinjiang Key Laboratory of Equine Breeding and Exercise Physiology, Urumqi 830052, China

**Keywords:** Junggar Bactrian camel, epididymis, mRNA, LncRNA, miRNA

## Abstract

The Junggar Bactrian camel, a primitive indigenous breed in China, exhibits low reproductive efficiency under natural grazing conditions. This is partly attributed to the development of the epididymis and the quality of semen, both of which directly affect reproductive performance. The epididymis is a key male reproductive organ responsible for sperm storage and transport. However, the gene expression profile of camel epididymal tissue remains poorly understood. In this study, we conducted whole-transcriptome sequencing of epididymal tissues from Junggar Bactrian camels before and after sexual maturity. A total of 683 differentially expressed mRNAs (DEmRNAs) were identified, including *TPM2*, *ITGA5*, *FASN*, and *ACP5*, of which 415 were upregulated and 268 were downregulated. Additionally, 260 differentially expressed long non-coding RNAs (DELncRNAs), including *LOC123611838*, *LOC105083505*, and *LOC123614702*, were identified, with 113 upregulated and 147 downregulated. An additional 11 differentially expressed microRNAs (DEmiRNAs), including *eca-miR-206* and *eca-miR-216a*, were also detected. Gene Ontology (GO) annotation and Kyoto Encyclopedia of Genes and Genomes (KEGG) pathway enrichment analyses revealed that key differentially expressed genes (DEGs), including *TPM2*, *ITGA5*, *DDIT4*, *FASN*, and *ACP5*, were mainly involved in pathways such as Cell Adhesion Molecules, Phospholipase D signaling, Cytokine–Cytokine Receptor Interaction, and Olfactory Transduction. This study presents a comprehensive whole-transcriptome analysis of the epididymis in Junggar Bactrian camels before and after sexual maturity, identifying key genes and regulatory pathways associated with epididymal development and reproductive function. These findings provide a theoretical foundation and valuable data for future research on reproductive performance and epididymal biology in Bactrian camels.

## Simple Summary

This study explored the genetic changes that occur in the epididymis of Bactrian camels as they mature sexually. By comparing camels before (3 years old) and after (5 years old) puberty, researchers discovered thousands of differences in gene activity, including, mRNA, protein-coding genes, long non-coding RNAs (LncRNAs), and microRNAs (miRNAs)—many of which were previously unknown. These genes play an important role in sperm storage, sperm transport, and hormone signaling. The key pathways involved in epididymal development were also identified, such as cell recognition, cell activation and signal transduction, cell proliferation and differentiation, cell stretching and movement, and other key signaling pathways. The findings provide new insights into camel reproduction and may help improve breeding programs for this economically important species.

## 1. Introduction

The Bactrian camel, a species adapted to extremely arid environments, has long been a subject of interest in the field of biological research due to its unique physiological mechanisms [1,2]. With strong stress resistance and drought tolerance, Bactrian camels are primarily distributed in China, Kazakhstan, and Russia, with smaller populations also found in countries such as Iran, Turkey, and India [3]. As seasonal breeders, Bactrian camels exhibit physiological characteristics such as long growth and gestation periods and low reproductive efficiency [4,5], leading to a gradual population decline and even the risk of extinction [6].

In male animals, the epididymis is an essential component of the reproductive system. It serves as the key site for sperm maturation, storage, and transport, and its regulatory mechanisms are critical in determining reproductive efficiency [7]. The epididymis is composed of a highly coiled and convoluted tubular structure, divided into several interconnected segments, each with distinct anatomical and functional characteristics [8]. It is a segmented organ consisting of three anatomically distinct regions: the caput (head), corpus (body), and cauda (tail), each with specialized biological functions [9]. Upon exiting the testis, sperm enter the epididymis in an immature state and gradually acquire motility and fertilizing capacity during their transit [10]. Approximately 90% of the fluid component is reabsorbed by epithelial cells in the caput, thereby increasing sperm concentration from testicular output [11]. Following maturation, 50–80% of the sperm are localized and stored in the cauda until ejaculation. In summary, the primary functions of the epididymis include sperm storage, transport, protection, and expulsion [12].

The application of whole-transcriptome sequencing has provided valuable insights into reproductive development, regulatory mechanisms, and sexual differentiation. By comparing transcriptomes of spermatogonial precursor cells and mature spermatozoa, key genes involved in spermatogenesis can be identified, offering a molecular perspective on sperm formation [13]. For instance, Shen et al. [14] showed that estrogen-related pathways may be enriched in different RNAs, and a total of 1081 DEmRNAs, 205 DELncRNAs, 54 DEcircRNAs, and 13 DEmiRNAs were upregulated in the winter estrus ovarian cortex (WAO) using whole transcriptome analysis of the ovarian cortex of Mongolian horses at different stages of estrus. A total of 1261 mRNAs, 90 LncRNAs, 29 circRNAs and 40 miRNAs were upregulated in the ovarian cortex (SDO) in summer estrus, which provided a theoretical basis for exploring the regulation of estrus in mare in different seasons. Similarly, Jin et al. [15] used single-cell RNA sequencing to study the cell composition, gene expression patterns, and regulatory effects during spermatogenesis and maturation in donkeys, analyzed the cell types and spatial distribution of donkey testes and epididymis, revealed the specific expression patterns of *CETN3* and *CDK1* in spermatogonia, and generated comprehensive transcriptional profiles at single-cell resolution. LncRNAs are non-coding RNAs more than 200 nucleotides in length, which play important roles in biological processes such as epigenetic regulation, cell cycle regulation, and regulation of cell differentiation [16]. LncRNAs regulate sperm viability by affecting testicular development as well as flagellar function and structure [17,18], and influence miRNA target gene expression, leading to biological process regulation, such as affecting testosterone production, cell proliferation, and antioxidant function of testicular mesenchymal cells [19]. Hasi et al. [20] found that whole transcriptome analysis of Bactrian camel testicular tissues showed that the LOC105072226-miR-362-3p-INHBB and LOC116153108-miR-140-5p-TLR2 network play key roles in regulating testicular development and sperm motility. To date, studies utilizing whole-transcriptome sequencing of epididymal tissue in Bactrian camels remain scarce. Therefore, by analyzing differentially expressed mRNAs and non-coding RNAs in the epididymal tissues of Junggar Bactrian camels before and after sexual maturity, this study elucidates molecular biological changes during epididymal development. These findings establish a foundation for future research on reproductive performance and developmental mechanisms in Bactrian camels.

## 2. Materials and Methods

### 2.1. Experimental Animals

This experiment was conducted in 2025 in Midong District, Urumqi City, Xinjiang. A total of eight Bactrian camels were selected, including four 3-year-old (pre-sexually mature) and four 5-year-old (post-sexually mature) individuals. The pre-sexual maturity group was designated as Group C (N = 4; 3 years old), and the post-sexual maturity group as Group E (N = 4; 5 years old) (see Supplementary Table S1). Epididymal tissue was collected and preserved in liquid nitrogen and 4% paraformaldehyde solution for further use.

### 2.2. RNA Extraction, Library Construction and Sequencing

Total RNA was extracted using the Trizol kit (Invitrogen, Carlsbad, CA, USA) according to the method provided by the manufacturer. RNA quality was assessed on an Agilent 2100 Bioanalyzer (Agilent Technologies, Palo Alto, CA, USA) and assayed using RNase-free agarose gel electrophoresis. After extraction of total RNA, two types of libraries were constructed: (1) de-ribosomal strand-specific libraries—rRNA was removed using a de-ribosomal kit, and the remaining RNA was fragmented for strand-specific library construction using the dUTP labeling method; (2) miRNA libraries—specific junctions are ligated at both ends of the RNA by ligation and amplified by reverse transcription, the PCR products are subjected to PAGE gels and recovered by cutting the gels (140 bp). Reverse transcription to cDNA was performed with the NEBNext Ultra RNA Library Prep Kit for Illumina (NEB #7530, New England Biolabs, Ipswich, MA, USA). Ligation reactions were purified with AMPure XP Beads (1.0X) (Creative Spectrum Technology, Beijing, China). Polymerase chain reaction (PCR) amplification was then performed. The cDNA library obtained was sequenced using Illumina Novaseq6000 (Ripple Gene Technology, Hangzhou, China) [21]. A total of 8 samples were studied in this experiment, each sample was divided into two copies, and a total of 16 cDNA libraries were obtained (see Supplementary Table S2 for detailed laboratory apparatus and equipment).

### 2.3. Data Quality Control and Identification of mRNAs, LncRNAs, and miRNAs

Filtering of Clean Reads, reads obtained from the sequencing machines includes raw reads containing adapters or low quality bases which will affect the following assembly and analysis. Thus, to produce high quality clean reads, reads were further filtered by fastp (version0.23.4). The parameters were as follows: (1) removing reads containing adapters; (2) removing reads containing more than 10% of unknown nucleotides (N); (3) removing low quality reads containing more than 50% of low quality (Q-value ≤ 20) bases. An index of the reference genome was built, and paired-end clean reads were mapped to the reference genome using HISAT2. 2.2.1 and other parameters set as a default. Transcripts less than 200 nt and those matching known mRNAs were discarded to identify LncRNA. Transcripts score less than 1 in CPC score and less than 0 in CNCI score were kept based on the CPC and CNCI programs. Potential LncRNAs are known to be contained in the remaining transcripts. To analyze the LncRNA, miRNA, and mRNA transcripts, the following criteria were used to identify significantly differential expressed transcripts: |log2fold change| ≥ 1.5 and |*p*| ≤ 0.05, with |*q*| ≤ 1.00 to correct the *p* value calculation [22]. In the current research, 41,421 mRNAs and 12,657 non-coding RNAs have been annotated in bactrian camels including (7231 LncRNAs, 2102 miscRNAs and 1933 tRNAs, etc.). This data was obtained from the Genome Data Viewer, Camelus bactrianus Annotation Report in NCBI. (Name:Ca_bactrianus_MBC_1.0, Refseq:GCF_000767855.1, Annotation Release:102, Release Date 20 December 2021).

### 2.4. Differential Expression Analysis

The mapped reads of each sample were assembled by using StringTie v2.2.1 in a reference-based approach. For each transcription region, a FPKM (fragment per kilobase of transcript per million mapped reads) value was calculated to quantify its expression abundance and variations.

### 2.5. GO and KEGG Enrichment Analysis

We performed gene set enrichment analysis using software GSEA. 4.4.0 and MSigDB. v2025.1.Hs to identify whether a set of genes in specific GO terms\KEGG pathways\Reactome pathways\DO terms shows significant differences in two groups. Briefly, we input gene expression matrix and rank genes by SignaltoNoise normalization method. Enrichment scores and p value was calculated in default parameters.

### 2.6. Quantitative Real-Time PCR

Extract total RNA from the epididymal sample, take a grinding tube, add 1ml of RNA extraction solution, add three 3 mm grinding beads, and pre-cool on ice. Take 5–20 mg of tissue and add it to the grinding tube. The grinder grinds well until there are no visible tissue blocks. Centrifuge at 12,000 rpm for 10 min at 4 °C to take the supernatant. Add 100 μL of chloroform substitute, invert the centrifuge tube for 15 s, mix well, and let stand for 3 min. Centrifuge at 12,000 rpm for 10 min at 4 °C. Transfer 400 μL of the supernatant to a new centrifuge tube, add 550 μL of isopropanol, and mix by inverting. Leave at −20 °C for 15 min. Centrifuge at 12,000 rpm at 4 °C for 10 min, and the white precipitate at the bottom of the tube is RNA. Aspirate the liquid, add 1 mL of 75% ethanol and mix to wash the pellet. Centrifuge at 12,000 rpm for 5min at 4 °C. Repeat steps 10–11) once. Suck the liquid clean, put the centrifuge tube on the clean table and blow for 3–5 min. Add 15 μL of RNA lysis solution to dissolve RNA. Use Nanodrop 2000 to detect RNA concentration and purity. After the instrument blank is zeroed, take 2.5 μL of the RNA solution to be tested on the detection base, put down the sample arm, and use the software on the computer to start the absorbance value detection. Dilute the RNA that is too high in an appropriate ratio to a final concentration of 200 ng/μL. Reverse transcription of total RNA into cDNA. Reverse transcription reaction (20 μL reaction set, reverse transcription kit catalog number G3337) was gently mixed and centrifuged, reverse transcription program was set up, and reverse transcription was completed on a common PCR instrument for RT-qPCR primer information. Take 0.1 mL of PCR plate and prepare the reaction system as follows, with 3 tubes of each reverse transcript product. After spotting the sample, the sealing film was completed with PCR sealing film and sealing instrument, and centrifugation was carried out with a microplate centrifuge. PCR amplification, which is performed on a real-time PCR instrument. All samples were subjected to 3 technical replicates. All lab equipment consumables (Supplementary Materials Table S3).

ΔΔCT method: A = CT (target gene, sample to be tested) − CT (internal standard gene, sample to be tested)

B = CT (target gene, control sample) − CT (internal standard gene, control sample)

K = A − B

Expression fold = 2 − K

### 2.7. Construction of the lncRNA-miRNA-mRNA Regulatory Network

We constructed a competitive endogenous RNA (ceRNA) network involving lncRNAs, miRNAs, and mRNA through the following steps: (1) The interactions between lncRNAs and miRNAs were assessed using miranda v3.3a. (2) Both miRDB and TargetScan v8.0 were employed to predict the associations between miRNAs and mRNAs. (3) By utilizing Cytoscape v3.9, we identified overlapping DElncRNAs and DEmiRNAs to generate a comprehensive ceRNA network encompassing lncRNA-miRNA-mRNA interactions.

### 2.8. Statistical Analysis

One-way ANOVA and multiple comparison analyses were performed using SPSS version 26.0 to compare the contents of epididymal tissue between the pre- and post-sexual maturity groups.

## 3. Results and Analysis

### 3.1. RNA-Seq Data Analysis

There were eight samples in this experiment and 16 cDNA libraries were obtained. The epididymal transcriptome generated approximately 1.34 billion high-quality reads (an average of 84,030,756.2 reads per library). In the whole transcriptome sequencing of the epididymis, the GC content ranged from 50.89% to 78.69%; Q20 content ranged from 89.95% to 98.5%; and Q30 content ranged from 80.76% to 95.56% (see Supplementary Table S4). Additionally, over 82.63% of the clean reads aligned with the reference genome.

### 3.2. Histological Observation of Epididymal Tissues Before and After Sexual Maturity

**Figure 1:**
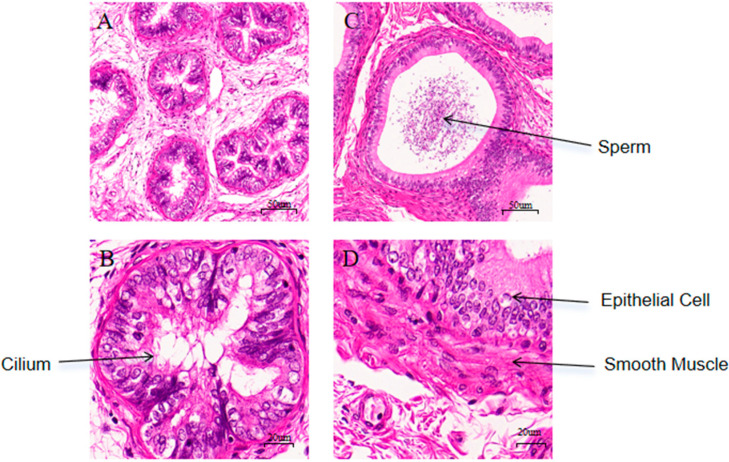
Histological observation of epididymal in before and after sexual maturity Bactrian camels. (**A**) Epididymal tissue section of before sexual maturity Bactrian camel (20× magnification); (**B**) Epididymal tissue section of before sexual maturity Bactrian camel (50× magnification); (**C**) Epididymal tissue section of after sexual maturity Bactrian camel (20× magnification); (**D**) Epididymal tissue section of after sexual maturity Bactrian camel (50× magnification).

HE staining of epididymal tissues from Group C (the pre-sexual maturity group) and Group E (the pre-sexual maturity group) showed clear visualization of sperm, epithelial cells, smooth muscle, and cilia. As shown in Figure 1A,C, the sperm count in the epididymis of Group E was significantly higher than that of Group C. As shown in Figure 1B,D, Group E had more epithelial cells and better-developed structures compared to Group C.

### 3.3. Expression Analysis of Samples from Epididymal Tissue Before and After Sexual Maturity

**Figure 2:**
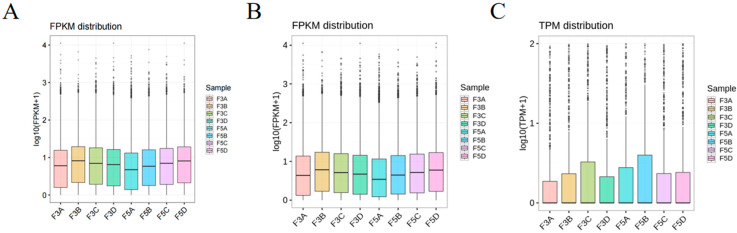
Box plots of transcriptome expression levels among different samples. (**A**) Box plot of mRNA expression levels; (**B**) Box plot of LncRNA expression levels; (**C**) Box plot of miRNA expression levels.

Based on the differential expression analysis of epididymal tissues from the pre-and post-sexual maturity Bactrian camels, the average transcript expression levels in Group C were found to be higher than those in Group E, as shown in Figure 2A,B. mRNA and LncRNA expression levels were higher in Group C, and inter-sample variation in gene expression was relatively small, indicating overall consistency in expression levels between before and after sexual maturity samples. Similarly, as shown in Figure 2C, the average transcript expression levels were higher in Group C compared to Group E, with miRNA expression levels also higher in Group C.

### 3.4. Differential Expression Analysis of Epididymal Tissues Before and After Sexual Maturity

**Figure 3:**
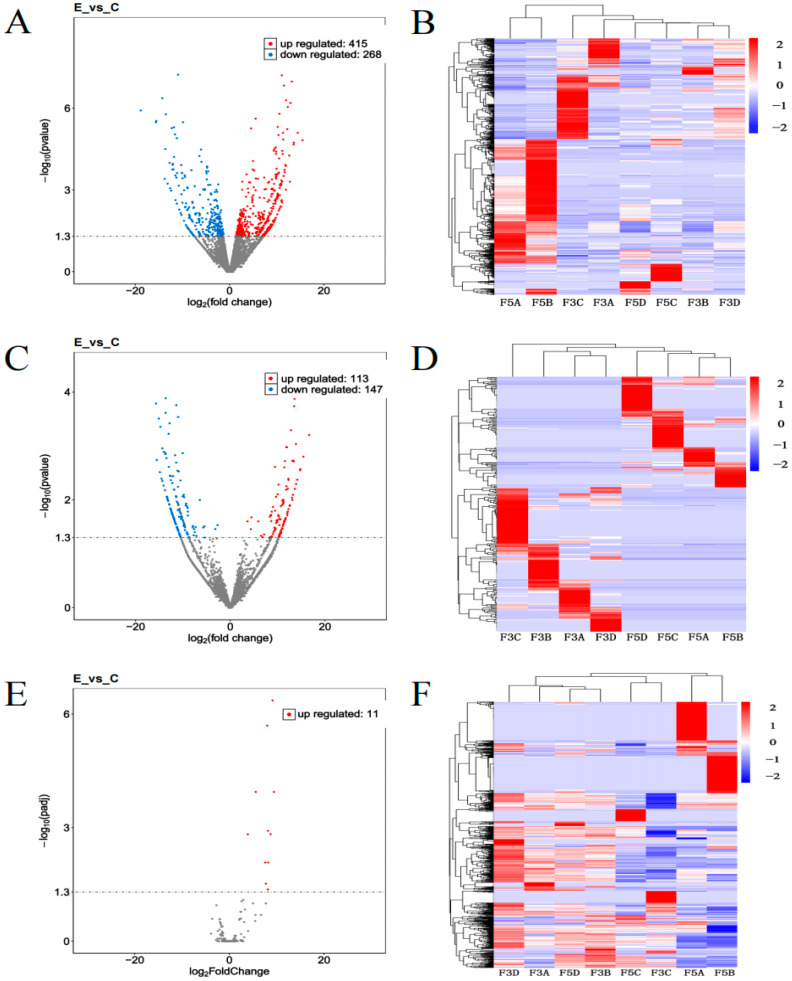
Volcano plots and cluster heatmaps of DEmRNAs, DELncRNAs, and DEmiRNAs. (**A**) Volcano plot of DEmRNAs; (**B**) Cluster analysis of DEmRNAs; (**C**) Volcano plot of DELncRNAs; (**D**) Cluster analysis of DELncRNAs; (**E**) Volcano plot of DEmiRNAs; (**F**) Cluster analysis of DEmiRNAs. Note: In (**A**,**C**,**E**), “up” and “down” indicate upregulated and downregulated gene expression in epididymal tissues of the before and after sexually mature Junggar Bactrian camels. In (**B**,**D**,**F**), the horizontal axis represents individual samples and the vertical axis represents expression levels; the color gradient from blue to red indicates increasing expression levels.

Differential expression analysis of epididymal tissues in before and after sexually mature Junggar Bactrian camels revealed the following: As shown in Figure 3A, 683 DEmRNAs such as *TPM2*, *ITGA5*, *FASN* and *ACP5* were screened, of which 415 DEmRNAs such as *TPM2* and *ITGA5* were upregulated, and 268 DEmRNAs such as *FASN* and *LUM* were downregulated. As shown in Figure 3B, 260 DELncRNAs such as *LOC123611838*, *LOC105083505* and *LOC123614702* were screened, of which 113 DELncRNAs such as *LOC123611838* and *LOC123614702* were upregulated, and 147 DELncRNAs such as *LOC105083505* and *LOC105076307* were downregulated. As shown in Figure 3C, 11 upregulated DEmiRNAs, such as *Eca-miR-206* and *Eca-miR-216a*, were screened. (see Supplementary Table S5). Cluster analysis (Figure 3B,D,F) showed high reproducibility of DEmRNAs, DELncRNAs, and DEmiRNAs in groups E and C in epididymal tissues in both the before and after sexual maturity groups, revealing significant differences between the two groups. Compared with LncRNA, mRNA has a higher overall expression level and has specific expression patterns in different tissues and cells.

### 3.5. GO Functional Annotation and KEGG Enrichment Analysis of Differentially Expressed Genes in the Epididymal Tissues of Before and After Sexual Maturity

#### 3.5.1. GO Annotation and KEGG Enrichment of DEmRNAs

**Figure 4:**
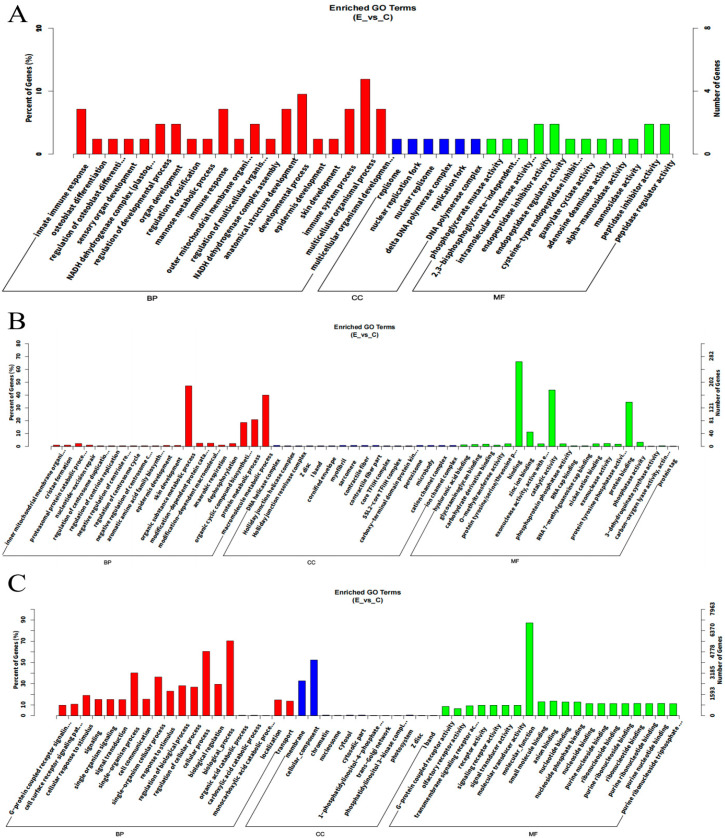
GO annotation of DEmRNAs, DELncRNAs, and DEmiRNA. (**A**) GO annotation of DEmRNAs; (**B**) GO annotation of DELncRNAs; (**C**) GO annotation of DEmiRNAs.

**Figure 5:**
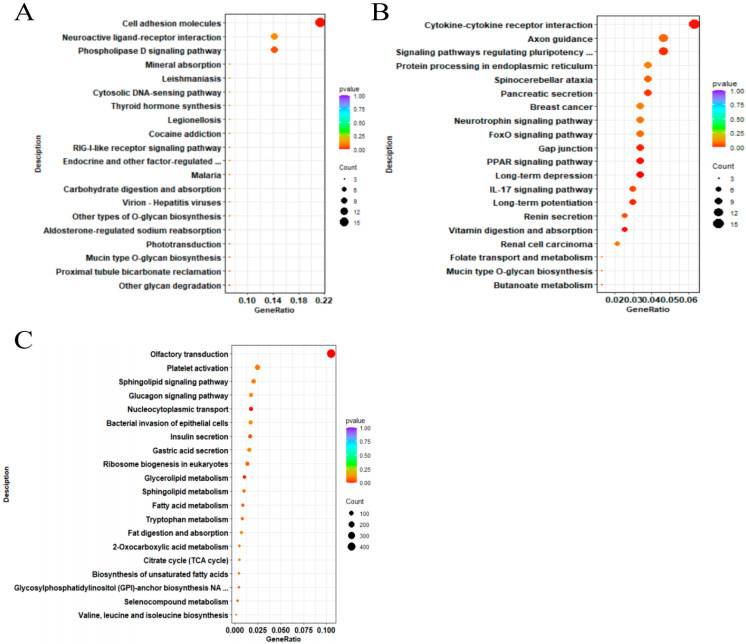
KEGG enrichment of DEmRNAs, DELncRNAs, and DEmiRNAs. (**A**) KEGG enrichment of DEmRNAs; (**B**) KEGG enrichment of DELncRNAs; (**C**) KEGG enrichment of DEmiRNAs.

As shown in Figure 4A, GO annotation revealed that DEmRNAs were mainly involved in terms including Multicellular Organismal Process (BP), Developmental Process (BP), and Innate Immune Response (BP). KEGG enrichment analysis (Figure 5A) showed that these DEmRNAs were significantly enriched in pathways such as Cell Adhesion Molecules, Phospholipase D, and Neuroactive Ligand–Receptor Interaction.

#### 3.5.2. GO Annotation and KEGG Enrichment of DELncRNAs

As shown in Figure 4B, GO annotation revealed that DELncRNAs were primarily involved in Binding (MF), Organic Substance Metabolic Process (BP), and Catalytic Activity (MF). KEGG enrichment analysis (Figure 5B) showed that these DELncRNAs were enriched in pathways such as Cytokine–Cytokine Receptor Interaction, Signaling Pathways Regulating Pluripotency of Stem Cells, and Axon Guidance.

#### 3.5.3. GO Annotation and KEGG Enrichment of DEmiRNAs

As shown in Figure 4C, GO annotation revealed that DEmiRNAs were mainly associated with terms including Molecular Function (MF), Biological Process (BP), and Cellular Process (BP). KEGG enrichment analysis (Figure 5C) indicated that the DEmiRNAs were enriched in pathways such as Olfactory Transduction, Platelet Activation, and the Sphingolipid Signaling Pathway (see Supplementary Table S6).

### 3.6. RT-qPCR Validation

**Figure 6:**
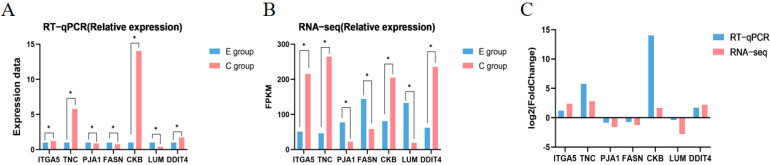
Validation of differentially expressed genes by RT-qPCR. (**A**) Relative expression of differentially expressed genes by RT-qPCR; (**B**) Relative expression of differentially expressed genes by RNA-seq; (**C**) Log_2_ fold-change comparison between RNA-seq and RT-qPCR for differentially expressed genes. Note: The * in the figure indicates a significant difference between the two groups (*p* < 0.05).

To verify the accuracy of transcriptome sequencing data, this study randomly selected seven differentially expressed genes—*ITGA5*, *TNC*, *FASN*, *CKB*, *LUM*, *PJA1*, and *DDIT4* for RT-qPCR validation. As shown in Figure 6, the expression levels of *ITGA5*, *TNC*, *CKB*, and *DDIT4* were significantly upregulated (*p* < 0.05); *FASN*, *LUM* and *PJA1* were significantly downregulated (*p* < 0.05). The expression trends of all genes were consistent between RT-qPCR and RNA-seq results, indicating that the sequencing data and expression profiles obtained in this study are reliable and can serve as a foundation for subsequent analyses.

### 3.7. Construction and Analysis of ceRNAs Regulatory Network

**Figure 7:**
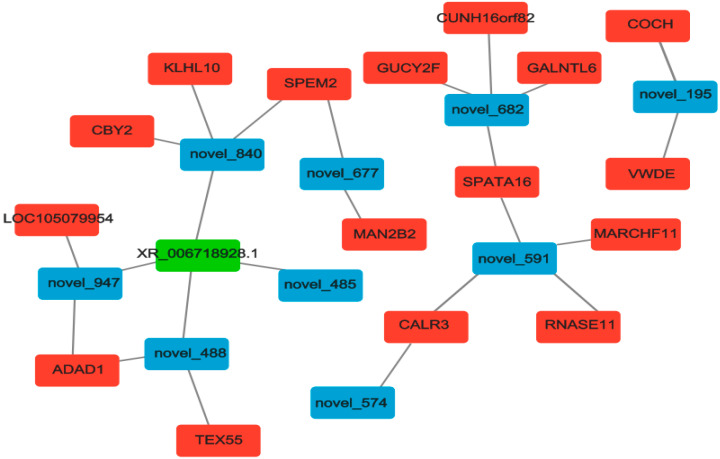
Network diagram of lncRNA–miRNA–mRNA interactions. Note: The shades of the colors indicate the lncRNAs (green), mRNAs (red), and miRNAs (blue).

Based on the regulatory relationship between DEmiRNA-DEmRNA and DEmiRNA-DElncRNA, as shown in Figure 7, we identified significantly differentially expressed lncRNAs and mRNAs that were co-regulated by the same miRNAs. This suggests that they may be key regulators of epididymal development and sperm maturation.

## 4. Discussion

The epididymis, as a vital reproductive organ, is responsible for functions such as sperm concentration, maturation (including the acquisition of sperm motility and fertilization capability), protection, and storage [23,24]. In males, sperm production begins when primitive germ cells differentiate into spermatogonial stem cells [25,26]. Spermatogenesis starts in the seminiferous tubules of the testis, where round spermatids mature into elongated sperm cells during the spermatogenic process [27]. The main function of the epididymis is to transport sperm smoothly from the convoluted seminiferous tubules of the testis to the vas deferens. This transport process typically takes around 10 days [10]. The human epididymis is approximately 3.8 cm in length [28], but its length varies among other male animals. Transcriptomic studies related to the epididymis have been applied in a variety of species, including cattle [29,30,31], pigs [32,33], sheep [34], chickens [35,36], and donkeys [37]. The head and body regions of the epididymis are mainly involved in sperm maturation, with sperm motility being the most prominent feature acquired after sperm undergo the maturation process [38]. The tail region of the epididymis is where sperm exhibit motility and acquire the ability to fertilize oocytes. After residing in the epididymis for one to two weeks, sperm reach the tail region, where they remain in a quiescent state until ejaculation [39,40]. Hasi et al. [20] sequenced the RNA in testicular tissue from pubertal and adult male camels, many expression genes and signaling pathways related to testicular development and spermatogenesis were discovered, such as positive regulation of cell proliferation, male germ cell nucleus, and male gonad development. Such as *TLR2*, *INHBB*, *STAT2* and *LHX6* are involved in the ceRNA regulatory network to inform our studies.

### 4.1. Differential Expression Analysis of mRNAs in the Epididymal Tissues of Bactrian Camels Before and After Sexual Maturity

In this study, 683 DEmRNAs were identified in epididymal tissues of Bactrian camels before and after sexual maturity, and functional annotation via GO and KEGG enrichment analyses was performed. The results showed that the majority of DEmRNAs were involved in various biological processes such as immunity, growth, metabolism, development, and reproduction. Thus, these DEmRNAs likely play important regulatory roles in the development of the epididymis during sexual maturation in Bactrian camels. KEGG enrichment analysis revealed that the DEmRNAs were primarily enriched in pathways including Cell Adhesion Molecules, Phospholipase D signaling, and Neuroactive Ligand–Receptor Interaction. Cajas et al. [41] found that the binding of sperm to the oviduct epithelium (mucosa) is mediated by interactions between cell adhesion molecules on the surfaces of sperm and epithelial cells. Nguyen et al. [42] reported that cadherins (CDHs), as important cell adhesion molecules, promote morphogenesis and the formation of tissue barriers by regulating cell movement, aggregation, and differentiation. In the testis, cadherins such as *CDH1*, *CDH2*, and *CDH3* are critical for gonadal development by facilitating the migration and subsequent aggregation of primordial germ cells with somatic cells. Mammalian sperm cannot fertilize oocytes immediately after ejaculation—capacitation is a complex process. In the Phospholipase D pathway, Grinshtain et al. [43] demonstrated that Phospholipase D and the Calmodulin Kinase II pathways prevent the acrosome reaction through two distinct mechanisms, both enhancing F-actin formation during capacitation. Protein tyrosine kinases mediate actin polymerization during capacitation, which is crucial for suppressing the acrosome reaction and thus enabling capacitation.

Based on the biological analysis of the DEmRNAs and their expression patterns, the candidate gene *TPM2* identified in this study is an important component of the cytoskeleton, primarily involved in regulating muscle contraction and maintaining cell morphology [44]. While research on *TPM2* has largely focused on muscle diseases (such as congenital myopathies) and cancer [45], its relevance to reproduction has gradually gained attention. The movement of the sperm tail (flagellum) relies on dynamic changes in the cytoskeleton, and *TPM2* may be involved in acrosome formation and maintaining flagellar structural stability during spermatogenesis. Joshi et al. [46] indicated that dynamic actin assembly is essential for sperm morphogenesis, and abnormal expression of *TPM2* could lead to sperm malformation or motility disorders (e.g., asthenozoospermia), resulting in male infertility. Certain mutations in *TPM2* have been associated with congenital myopathies and may lead to muscle function abnormalities in offspring through inheritance. These mutations might impair muscle development or hormone regulation in the reproductive system, thereby indirectly reducing reproductive capacity. Wu et al. [47] found that *TPM2* can slow the progression of prostate cancer (PCa) by blocking *PDLIM7*-mediated nuclear translocation of *YAP1*, suggesting that modulation of *TPM2* expression or function may be a potential therapeutic strategy to reduce PCa proliferation and prevent the progression to castration-resistant prostate cancer (CRPC). Jakub Kulus et al. [48] also identified *TPM2* as a key regulator in porcine ovarian follicular granulosa cells, functioning in cytoskeletal assembly, intracellular organelle organization, and cell division regulation.

*DDIT4* may play a role in maintaining genome stability by regulating cell cycle arrest or apoptosis in response to DNA damage in germ cells. Wang et al. [49] found that exposure to diisononyl phthalate (DINP) can induce autophagy in ovarian granulosa cells while increasing mRNA and protein levels of *DDIT4*. Furthermore, overexpression of *DDIT4* has been shown to induce autophagy in ovarian granulosa cells, while knockout of *DDIT4* inhibited DINP-induced autophagy, indicating that *DDIT4* plays a key role in this process. Kong et al. [50] reported that circ*DDIT4* is downregulated in PCa and acts as a tumor suppressor during PCa progression. Therefore, *DDIT4* may participate in critical regulatory processes in the reproductive system.

### 4.2. Differential Expression Analysis of LncRNAs in the Epididymal Tissues of Bactrian Camels Before and After Sexual Maturity

LncRNAs play an important regulatory role in epididymal development, affecting physiological processes such as cell proliferation, differentiation, and hormone secretion. Lewandowski et al. [51] demonstrated in their mouse knockout experiments that the deletion of *Tug1* in male mice results in male infertility. KEGG enrichment analysis of the target genes of DELncRNAs revealed that numerous DELncRNAs are enriched in signaling pathways related to epididymal development, spermatogenesis, and hormone secretion, such as the Cytokine–Cytokine Receptor Interaction, Signaling Pathways Regulating Pluripotency of Stem Cells, and Axon Guidance pathways. And the signaling pathways such as Cytokine–Cytokine Receptor Interaction and Signaling Pathways Regulating Pluripotency Of Stem Cells were consistent with the signaling pathways that were partially enriched to mRNAs in this study, indicating that mRNAs and LncRNAs partially overlapped in the relevant functional annotation and pathway enrichment, serving as co-expression. For example, Hitit et al. [52] reported that LncRNAs possess higher and more stable biological structures, and by interacting with mRNAs, proteins, and other small non-coding RNAs (sncRNAs), they regulate gene expression at multiple levels. They identified DELncRNAs in ram sperm and their expression processes related to reproductive performance. Callaghan et al. [53] demonstrated that the reproductive condition of boars significantly impacts the endometrial changes in pregnant cows, and the Cytokine–Cytokine Receptor Interaction pathway mediates the differences in gene expression between bulls with high and low reproductive performance. In conclusion, this study identifies DELncRNAs and their associated pathways in the epididymal tissues of Bactrian camels before and after sexual maturity, which may serve as important candidate factors for improving Bactrian camel reproductive performance in future studies.

### 4.3. Differential Expression Analysis of miRNAs in the Epididymal Tissues of Bactrian Camels Before and After Sexual Maturity

It has been reported that miRNAs play a critical role in male epididymal development. Chen et al. [54] found that a decrease in oxidative phosphorylation levels in the human epididymis, along with chronic hypoxia, may impair sperm production. In a study on epididymal inflammation induced by lipopolysaccharides (LPS), leukocyte infiltration and fibrosis were observed in the tail region of mice, and these inflammatory responses could be eliminated in *TNFA* knockout mice. To explore the correlation between these DEmiRNAs and the epididymal tissues of Bactrian camels before and after sexual maturity, KEGG analysis of their target genes revealed significant enrichment in pathways such as Olfactory Transduction, Platelet Activation, and Sphingolipid Signaling. Karuthadurai et al. [55] found that the expression changes in transcripts is associated with the olfactory receptor pathway in bulls with poor semen quality, indicating a potential correlation between the olfactory pathway and semen quality. For instance, G Protein-Coupled Receptors (GPCRs) are involved in sensory perception, chemical stimulus detection, olfactory perception, signal transduction, and synaptic transmission, significantly disrupting sperm motility in poor-quality semen. The Platelet Activation pathway plays a crucial role in biological processes such as cell proliferation, differentiation, survival, and migration. Wu et al. [56] showed that platelet-activating factor (PAF) modulates sperm capacitation, acrosome reaction, and fertilization potential. Specifically, PAF upregulates extracellular signal-regulated kinases (ERK) and tyrosine phosphorylation levels in sperm, suggesting that PAF may actively participate in regulating the acrosome reaction through interaction with ERK. The Sphingolipid Signaling pathway is involved in various physiological processes. Ji et al. [57] observed that excessive accumulation of Reactive Oxygen Species (ROS) during semen preservation in Lake sheep disrupts sperm antioxidant homeostasis. Thus, adding appropriate concentrations of curcumin to sheep semen can affect metabolites such as Sphingosine-1-Phosphate and Plant Sphingosine, thereby inhibiting ROS production and prolonging semen storage time.

Based on the unique regulatory relationships between DELncRNAs, DEmiRNAs, and DEmRNAs, we constructed ceRNA regulatory networks in both the testis and epididymis. This study suggests that XR-006718928.1-novel-840-SPEM2 and XR-006718928.1-novel-488-TEX55 may have the potential to promote epididymal development and sperm maturation. As predicted, novel-840 targets XR-006718928.1 and *SPEM2*, a member of the *SPEM* family of genes that all contain unknown functional domains and are highly conserved throughout evolution. Li et al. [58] that *SPEM* family member 2 (*Spem2*), as a novel testis-enriched gene, is essential for spermiogenesis and male fertility. In vivo fertilization assays have shown that *Spem2*-null sperm are unable to fertilize oocytes, possibly due to their impaired ability to migrate from the uterus into the oviduct. *TEX55* (TSCPA, C3orf30 in humans), which was proposed to possess an A-kinase anchoring protein (AKAP) activity [59]. Gangwar et al. [60] showed that among the genetic variants identified using genome-wide SNP chip array data to significantly affect important reproductive traits in Vrindavani cattle, *TEX55*, *ITGB5*, *ADAM2* and *UPK1B* are new potential candidate genes related to reproductive traits and can be used as candidate biomarkers in animal improvement programs to improve the reproductive performance.

## 5. Conclusions

The results of this study indicate that DEGs such as *TPM2*, *DDIT4*, *ITGA5* and *LOC105083505* play active roles in signaling pathways such as Cell Adhesion Molecules, PI3K/Akt and Olfactory Transduction, and these DEGs may interact with XR-006718928.1 -novel-840-SPEM2 and XR-006718928.1-novel-488-TEX55 to regulate epididymal development and promote sperm maturation in male Junggar Bactrian camels. Due to the lack of functional validation experiments in this study, our group is actively culturing primary cells from Bactrian camel epididymis to further complete the functional validation of the cells.

## Data Availability

The data presented in this study are openly available in BioProject with reference number PRJNA1249394 (Table S1); Laboratory instruments and equipment (Table S2); Primers used in this study (Table S3); Clean reads and alignment results to the reference genome from transcriptome sequencing (Table S4); Differentially expressed mRNA, LncRNA, MiRNA (Table S5); GO and KEGG enrichment information of differentially expressed mRNA, LncRNA, and MiRNA (Table S6) have been deposited at Figshare and available at: https://doi.org/10.6084/m9.figshare.28852421 (accessed on 11 April 2025).

## Supplementary Materials

The following supporting information can be downloaded at: https://www.mdpi.com/article/10.3390/biology14070760/s1, Table S1. Sample grouping information; Table S2. Laboratory instruments and equipment; Table S3. Primers used in this study; Table S4. Clean reads and alignment results to the reference genome from transcriptome sequencing; Table S5. Differentially expressed mRNA, LncRNA, MiRNA; Table S6. GO and KEGG enrichment information of differentially expressed mRNA, LncRNA, and MiRNA.
